# Iron deficiency in blood donors

**DOI:** 10.1590/S1516-31802001000400003

**Published:** 2001-07-07

**Authors:** Rodolfo Delfini Cançado, Carlos Sérgio Chiattone, Fausto Forin Alonso, Dante Mário Langhi, Rita de Cássia Silva Alves

**Keywords:** Iron deficiency, Blood donor, Serum ferritin, Deficiência de ferro, Doador de sangue, Ferritina sérica

## Abstract

**CONTEXT:**

Blood donation results in a substantial loss of iron (200 to 250 mg) at each bleeding procedure (425 to 475 ml) and subsequent mobilization of iron from body stores. Recent reports have shown that body iron reserves generally are small and iron depletion is more frequent in blood donors than in non-donors.

**OBJECTIVE:**

The aim of this study was to evaluate the frequency of iron deficiency in blood donors and to establish the frequency of iron deficiency in blood donors according to sex, whether they were first-time or multi-time donors, and the frequency of donations per year.

**DESIGN:**

From September 20 to October 5, 1999, three hundred blood donors from Santa Casa Hemocenter of São Paulo were studied.

**DIAGNOSTIC TESTS:**

Using a combination of biochemical measurements of iron status: serum iron, total iron-binding capacity, transferrin saturation index, serum ferritin and the erythrocyte indices.

**RESULTS:**

The frequency of iron deficiency in blood donors was 11.0%, of whom 5.5% (13/237) were male and 31.7% (20/63) female donors. The frequency of iron deficiency was higher in multi-time blood donors than in first-time blood donors, for male blood donors (7.6% versus 0.0%, P < 0.05) and female ones (41.5% versus 18.5%, P < 0.05). The frequency of iron deficiency found was higher among the male blood donors with three or more donations per year (P < 0.05) and among the female blood donors with two or more donations per year (P < 0.05).

**CONCLUSIONS:**

We conclude that blood donation is a very important factor for iron deficiency in blood donors, particularly in multi-time donors and especially in female donors. The high frequency of blood donors with iron deficiency found in this study suggests a need for a more accurate laboratory trial, as hemoglobin or hematocrit measurement alone is not sufficient for detecting and excluding blood donors with iron deficiency without anemia.

## RESUMO

**INTRODUÇÃO:**

A doação de sangue causa redução substancial na quantidade de ferro do organismo (cada unidade de sangue retirada [425 a 475 ml] contém 200 a 250 mg de ferro) com conseqüente mobilização do ferro de depósito. Recentes estudos têm demonstrado que as reservas de ferro do organismo são pequenas, e sua deficiência é mais freqüente nos doadores de sangue que nos indivíduos não-doadores de sangue.

**OBJETIVO:**

Avaliar a freqüência da deficiência de ferro em doadores de sangue do Hemocentro da Santa Casa de São Paulo segundo o sexo, o tipo de doador e a freqüência de doações realizadas nos últimos 12 meses.

**PARTICIPANTES:**

No período de 20 de setembro a 5 de outubro de 1999 foram estudados 300 doadores de sangue.

**TESTES DIAGNÓSTICOS:**

Utilizamos a determinação das dosagens do ferro sérico, da capacidade total de ligação do ferro, do índice de saturação da transferrina, da ferritina sérica e dos índices eritrocitários.

**RESULTADOS:**

A freqüência de doadores de sangue que apresentavam deficiência de ferro observada foi de 11, 0 % (33/300), sendo 5, 5% (13/237) nos doadores do sexo masculino e de 31, 7% (20/63) nos doadores do sexo feminino. Para as mulheres, 18, 5% delas já apresentavam deficiência de ferro à primeira doação de sangue, e para as doadoras freqüentes foi 41, 5%. Entre os homens, nenhum doador apresentava deficiência de ferro à primeira doação de sangue e, para os doadores freqüentes, a deficiência de ferro foi de 7, 6%. A freqüência observada foi maior para os doadores com três ou mais doações nos últimos 12 meses (P < 0,05) e para as doadoras com duas ou mais doações nos últimos 12 meses (P < 0,05).

**CONCLUSÕES:**

A doação de sangue constituise numa causa importante de deficiência de ferro em doadores de sangue, particularmente nos doadores freqüentes do sexo feminino. A elevada freqüência de doadores de sangue com deficiência de ferro observada nesse estudo sugere a necessidade de uma triagem laboratorial mais acurada, uma vez que a determinação isolada da hemoglobina ou do hematócrito não é suficiente para detectar e excluir os doadores com deficiência de ferro ainda sem anemia.

## INTRODUCTION

The general impact of blood donation on iron status has been studied since the late 1970's.^1-5^ Blood donation results in a substantial (200 to 250 mg) loss of iron at each bleeding procedure (425 to 475 ml) and subsequent mobilization of iron from body stores. Recent reports have shown that body iron reserves generally are small and iron depletion is more frequent in blood donors than in non-donors.^4^^,^^6^

An inverse correlation exists between body iron stores and absorbed iron. As body iron stores decrease, iron absorption increases. With continued iron loss, an individual either reaches equilibrium at a lower concentration of iron stores or becomes iron-depleted, eventually developing iron-deficient erythropoiesis and anemia.^6^

In the majority of blood banks, hemoglobin (Hb) and/or hematocrit measurements are used as a screening test for the ability to donate blood even though iron stores may be depleted in donors with Hb values above the arbitrarily defined limit for anemia.^7^

It is known that iron deficient anemia is the last stage of iron-deficiency and it is evident that hemoglobin measurement alone is inadequate for detecting blood donors with iron deficiency without anemia.^8^^,^^9^

Recent literature has suggested that serum ferritin levels appear to be a reliable indicator for body iron stores that can be mobilized and provide reliable measurements for determining iron deficiency at an early stage. Serum ferritin is directly proportional to body iron stores and concentration <12 ng/ml reflects an iron-depleted state.^9-13^

The aim of this study was to evaluate the frequency of iron deficiency in Santa Casa Hemocenter blood donors and to establish the frequency of iron deficiency in blood donors according to sex, whether they were first-time or multi-time donors, and the blood donation frequency per year.

## METHODS

From September 20 to October 5, 1999, three hundred blood donors were studied using a combination of biochemical measures of iron status: serum iron (Bayer Company), total iron-binding capacity (TIBC) (Labtest Company), transferrin saturation index (TSI) [(serum iron/ CTLF) x100], serum ferritin (determined by the enzyme immunoassay method, Abbott Laboratories) and the erythrocyte indices (Cell-Dyn, model 3000, Abbott Laboratories).

Approximately 450 ml of blood was drawn at each phlebotomy and blood samples were taken at the end of the procedure.

According to the Brazilian Government requirement for blood donation^14^ we included in this study only donors with standard values of hemoglobin (≥12 g/dl for women and ≥13 g/dl for men) and/or hematocrit (38% for women and 40% for men)

Iron-depleted donors were defined by serum ferritin values below 12 ng/ml and TSI greater than or equal to 16%; iron-deficient erythropoiesis when serum ferritin values were less than 12 ng/ml and TSI less than 16%; and iron-deficiency anemia when serum ferritin values were less than 12 ng/ml and TSI less than 16%, and hemoglobin less than 13 g/dl (for men) or 12 g/dl (for women). Iron deficiency includes iron-depleted donors and iron-deficient erythropoiesis donors.

In statistical analysis, differences were evaluated by the Mann-Whitney rank sum test and the Student "t" test, and correlations using Spearman's rank correlation coefficient.^15^ A "P" value less than 0.05 was considered as statistically significant. All the statistical analyses were performed on the software SPSS, version PC+.

## RESULTS

The characteristics of the 300 blood donors that were studied were the age in years (mean 33.0; median 32.0; range 18 to 60); the sex, with 79% being male (237/300); and the color, with 35% being non-white (104/300). The number of first-time blood donors was 94 (31.3%), and there were 206 (68.7%) multi-time blood donors. Of the latter, 133 (64.5%) were considered repeat donors (who had donated one or more times per year) and 73 (35.5%) sporadic donors.

The frequency of iron deficiency in blood donors was 11.0%, of whom 5.5% (13/237) were male and 31.7% (20/63) female donors. The frequency of iron deficiency was higher in multi-time blood donors than in first-time blood donors, for male blood donors (7.6% versus 0%, P < 0.05) and female ones (41.5% versus 18.5%, P < 0.05) (Table 1).

**Table 1:** Frequency of iron deficiency in blood donors according to sex and type of donor

Sex	Male n = 237 (79%)		Female n = 63 (21%)	
Type of Donor	First-time (n = 67)	Multi-time (n = 170)	First-time (n = 27)	Multi-time (n = 36)
Iron Deficiency, n (%)	0 (0.0)	13 (7.6) *	5 (18.5)	15 (41.5) *
No Iron Deficiency	67 (100.0)	157 (92.3)	22 (81.5)	21 (58.5)

* P < 0.05.

**Figure 1:**
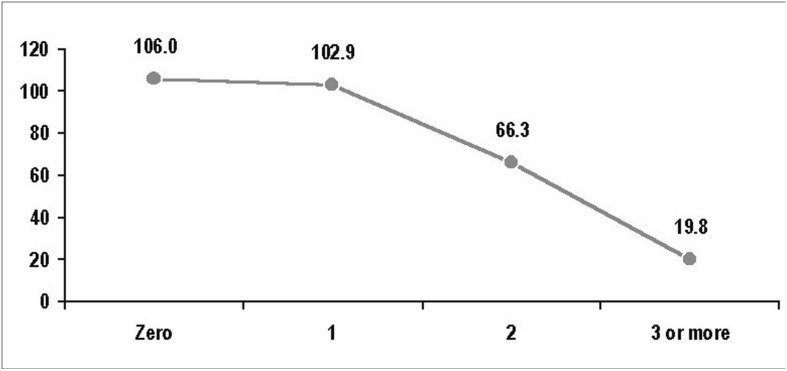
Mean ferritin serum level observed in the male blood donors according to the blood donation frequency in the last 12 months.

**Figure 2:**
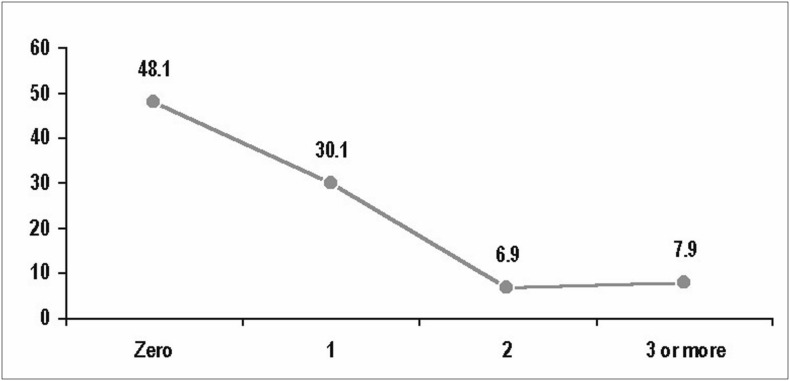
Mean ferritin serum level observed in the female blood donors according to the blood donation frequency in the last 12 months.

Figures 1 and 2 show the mean ferritin serum level observed in the male and female blood donors, respectively, according to the blood donation frequency in the last 12 months.

The frequency of iron deficiency found was higher in the multi-time blood donors. This difference was statistically significant among the male blood donors with three or more donations per year (P < 0.05) and among the female blood donors with two or more donations per year (P < 0.05) (Table 2).

**Table 2:** Distribution of serum ferritin, transferrin saturation index (TSI) and the frequency of iron deficiency in male blood donors according to the blood donation frequency in the last 12 months

Blood Donation Frequency in the last 12 months	N	Male Blood Donors: Iron Deficiency n (%)	N	Female Blood Donors: Iron Deficiency n (%)
				
Zero	127	1 (0.8)	9	9 (22.5)
1	94	2 (2.1)	7	7 (36.9) *
2	15	9 (60.0) *	2	2 (100.0)
^3^ 3	1	1 (100.0)	2	2 (100.0)
**Total**	**237**	**13 (5.5)**	**63**	**20 (31.7)**

N = number of blood donors in each group;

(*) P < 0.05.

## DISCUSSION

Iron is a vitally important element in human metabolism. It has a central role in erythropoiesis and is also involved in many other intracellular processes in all the tissues of the body.^16^^,^^17^

The potential for an individual donor to give blood without developing iron deficiency anemia displayed wide variation, probably due to differences in nutritional iron intake, the prevalence of iron deficiency in the particular population, the menstrual iron loss in females, the frequency of blood donation and the use of supplemental iron, as well as the capacity to absorb iron.^5^^,^^6^^,^^18^

Recent reports have shown that the frequency of iron deficiency is high in blood donors (1.8% to 8.4% in males and 4.5% to 34.8% in females), and more dependent on the frequency of donations than on the accumulated number of donations, ^5^^,^^6^^,^^18-24^ as we also found in the present study.

The only known significant disadvantage of blood donation is the potential risk for iron deficiency. Therefore it seems reasonable to secure adequate iron reserves in the donor population in order to maintain an appropriate donation potential and to avoid possible non-hematological side-effects of iron deficiency, i.e. changes in immune function, energy metabolism and work performance.^8^^,^^19^

## CONCLUSIONS

We conclude that blood donation has a profound influence on iron stores and is a very important factor for iron deficiency in blood donors, particularly in multi-time donors and, especially in female donors. The high frequency of blood donors with iron deficiency found in this study suggests a need for a more accurate laboratory trial, since hemoglobin or hematocrit measurement alone is not sufficient for detecting and excluding blood donors with iron deficiency without anemia.

## References

[1] Lieden G (1973). Iron state in regular blood donors. Scand J Haematol.

[2] Finch CA, Cook JD, Labbe RF, Cuala M (1977). Effect of blood donation on iron stores as evaluated by serum ferritin. Blood.

[3] Skikne BS, Cook JD (1981). Serum ferritin in the evaluation of iron status. Lab. Management.

[4] Agha F, Khan RA (1989). Ferritin levels in professional blood donors. JAMA.

[5] Milman N, Kirchhoff M (1991). Influence of blood donation on iron stores assessed by serum ferritin and haemoglobin in a population survey of 1433 Danish males. Eur J Haematol.

[6] Milman N, Sondergaard M (1984). Iron stores in male blood donors evaluated by serum ferritin. Transfusion.

[7] Chueca MP, Galar GM, Ardanaz MF, Zabalegui A, Muruzábal L, Munhoz A (1995). La hemoglobina en la selección de hemodonación. Sangre.

[8] Baynes RD, Brock JH, Halliday JW, Pippard MJ, Powell LW (1994). Iron deficiency. Iron metabolism in health disease.

[9] Worwood M, Brock JH, Halliday JW, Pippard MJ, Powell LW (1994). Laboratory determination of iron status. Iron metabolism in health and disease.

[10] Addison GM, Beamish MR, Hales CN (1972). An immunoradiometric assay for ferritin in the serum of normal subjects and patients with iron deficiency and iron overload. J Clin Pathol.

[11] Miles LEM, Lipschitz DA, Bieber CP, Cook JD (1974). Measurement of serum ferritin by a 2-site immunoradiometric assay. Anal Biochem.

[12] Cook JD, Lipschitz DA, Miles LA, Finch CA (1974). Serum ferritin as a measure of iron stores in normal subjects. Am J Clin Nutr.

[13] Lipschitz DA, Cook JD, Finch CA (1974). A clinical evaluation of ferritin as an index on iron store. N Engl J Med.

[14] Brasil. Ministério da Saúde (1993). Gabinete do Ministro. – Portaria n. 1376 de 19 de novembro de 1993. Aprova alterações na Portaria n. 721/GM, de 09.08.89, que aprova Normas Técnicas para coleta, processamento e transfusão de sangue, componentes e derivados, e dá outras providências. Diário Oficial da União.

[15] Rosner B (1986). Fundamentals of biostatistics.

[16] Aisen P (1982). Current concepts in iron metabolism. Clin Haematol.

[17] Brittenham GM, Hoffman R, Bens E, Shatiil S, Furie B, Choen H (1991). Disorders of iron metabolism: deficiency and overload. Hematology: basic principles and practice.

[18] Jacob RA, Sandstead HH, Klevay LM, Johnson LK (1980). Utility of serum ferritin as a measure of iron deficiency in normal males undergoing repetitive phlebotomy. Blood.

[19] Guerra CCC (1988). Carência de ferro. Bol Soc Hemato.

[20] Romero MS, Puente F, Abós MD, Gutiérrez M (1989). Incidencia de ferropenia en un colectivo de 922 candidatos altruistas a donantes de sangre. Sangre.

[21] Lamas MC, Pérez-Lanzac JCL, Arrojo IP, Gordo RS, Christensen EA, Font ES (1994). Determinación de ferritina sérica: Consideraciones para evitar ferropenia inducida en donantes de sangre. Sangre.

[22] Milman N, Pedersen NS, Visfeldt J (1983). Serum ferritin concentrations and iron stores in normal subjects. Serum ferritin in healthy Danes: relation to marrow haemosiderin iron stores. Dan Med Bull.

[23] Gualandro SFM, Cliquet MG, Silveira PAA (1999). Deficiência de ferro em doadoras de sangue da Fundação Pró-sangue/ Hemocentro de São Paulo. Ser Monogr Esc Bras Hematol.

[24] Birgegard G, Hogman C, Killander A, Wide L (1978). Serum ferritin levels in male blood donors. Relation to number of phlebotomies and iron supplementation. Vox Sang.

